# Screening and identification of microRNAs mediating cartilage endplate degeneration in human intervertebral disks

**DOI:** 10.3389/fmed.2024.1446294

**Published:** 2024-10-08

**Authors:** Bei Jiang, Chong Bai, Jie Pan, Bin Shen, Lijun Li

**Affiliations:** ^1^Department of Spine Surgery, Zhejiang Rongjun Hospital, School of Medicine, Jiaxing University, Jiaxing, China; ^2^Department of Spine Surgery, Shanghai East Hospital, School of Medicine, Tongji University, Shanghai, China

**Keywords:** intervertebral disk degeneration, cartilage endplate, microRNA, DEGs, hsa-miR-25-3p, hsa-miR-345-5p

## Abstract

**Objective:**

This study aimed to discover micro-ribonucleic acids (microRNAs) involved in the degeneration of cartilage endplates through next-generation sequencing and lay the foundation for further research.

**Methods:**

The cartilage endplate was obtained from patients who underwent interbody fusion surgery at the Department of Spine Surgery, Shanghai East Hospital Affiliated to Tongji University, from 1 January 2020 to 1 January 2023. Total RNA was extracted from the cartilage endplate tissue. Discover differential genes through NGS. To annotate gene functions, all target genes were aligned against the Kyoto Encyclopedia of Genes (KEGG) and Gene Ontology (GO) databases. The GO enrichment and KEGG enrichment analyses of target genes were performed using phyper, a function of R. The *p*-value was corrected using the Bonferroni method, and a corrected *p*-value of ≤0.05 was taken as the threshold. GO terms or KEGG terms fulfilling this condition were defined as significantly enriched terms. The screened miRNAs and their target protein were verified *in vitro* using quantitative polymerase chain reaction (qPCR) and Western blotting (WB).

**Results:**

RNA was extracted from normal and degenerated cartilage endplate tissues for NGS. Eight downregulated differentially expressed genes (DEGs) and 22 upregulated DEGs were found. The KEGG pathway analysis of these target genes revealed that differential microRNAs and target genes were enriched in different signaling pathways, and the regulated signaling pathways were mainly mitochondrial autophagy and autophagy. The qPCR results demonstrated a significant upregulation of miR-25-3p and miR-345-5p in degenerative cartilage endplate tissues (*p* ≤ 0.001). Western blot analysis revealed that BRD4 exhibited a marked increase in protein expression levels in degenerative cartilage endplate tissues (*p* ≤ 0.0001), while BECN1 showed a significant decrease in protein expression levels within these samples (*p* ≤ 0.0001).

**Conclusion:**

We found that DEG hsa-miR-25-3p and hsa-miR-345-5p can be used as diagnostic and therapeutic targets for IDD. The significant target proteins of miR-25-3p and miR-345-5p were BRD4 and BECN1, respectively.

## 1 Introduction

Degenerative disk diseases have become a serious public health problem worldwide due to their high prevalence and high cost (1, 2). However, the etiology and specific mechanism of intervertebral disk degeneration are still unclear. More and more studies have confirmed that the degeneration of the cartilage endplate is not only related to the degeneration of the intervertebral disk but also the initial factor of the degeneration of the intervertebral disk. Roberts et al. (3) found that calcification in the deep layer of the extra-endplate region in mice significantly precedes the degeneration of the nucleus pulposus *in vivo*. Zehra et al. (4) found that cartilage endplate degeneration can lead to nucleus pulposus degeneration, and the thickness of cartilage endplate calcification is positively correlated with the degree of intervertebral disk degeneration by comparing the degree of intervertebral disk degeneration and cartilage endplate degeneration in rats.

Micro-ribonucleic acid (microRNA) is a class of endogenous, non-coding single-stranded ribonucleic acid (RNA) molecules approximately 21–25 nucleotides in length. The latest study found that miR-34a-5p can enhance the resistance of nucleus pulposus cells to stress compression injury by inhibiting Sirtuin1 (SIRT1) (5). The downstream target of miR-31-5p is matrix metalloproteinase-3 (MMP3), and its downregulation can promote the degradation of the extracellular matrix and lead to intervertebral disk degeneration (6). The increased expression level of miR-24-3p in human degenerated nucleus pulposus cells can induce the apoptosis of nucleus pulposus cells, which is positively correlated with the degree of intervertebral disk degeneration (7). In addition, previous studies have found that the knockdown of related microRNAs can reduce the effect of inflammation on nucleus pulposus cells (8, 9). However, the current research mainly focuses on the nucleus pulposus tissue of the intervertebral disk, and there are few related studies on the cartilaginous endplate.

Therefore, the purpose of this study was to search for differentially expressed microRNA in the degenerative human cartilage endplate through high-throughput sequencing, analyze and screen its target genes and main signaling pathways, and provide new targets for the diagnosis and treatment of IDD.

## 2 Methods

### 2.1 Collection of samples

The cartilage endplate samples used in this study were obtained from patients who underwent interbody fusion surgery at the Department of Spine Surgery, Shanghai East Hospital Affiliated to Tongji University, from 1 January 2021 to 1 January 2023. The cartilage endplates were isolated by the surgeon during the operation and then placed in liquid nitrogen at-80°C for preservation. The inclusion criteria were as follows: patients with spinal trauma or spinal degenerative diseases. The exclusion criteria were as follows: patients who underwent surgery for spinal tumors or tuberculosis and patients with systemic diseases. All patients have signed the relevant informed consent forms. The study has been approved by the Ethics Committee of Shanghai East Hospital Affiliated to Tongji University. A total of six cartilage endplate tissue samples, including three normal cartilage endplate tissues and three degenerated cartilage endplate tissues, were judged based on preoperative MRI images and intraoperative conditions. Three cases were diagnosed with hyperextension injuries of the cervical spine (HEICS) and the other three with disk herniation (DH). The degree of disk degeneration was assessed using the modified Pfirrmann disk degeneration grade (Table 1).

**Table 1:** The details of cartilage endplate tissue samples.

Simples	Age (Year)	Sex	BMI	Level	Pre-operative diagnosis	Modified pfirrmann disk degeneration grade
1	65	M	24.2	C5-C6	DH	6
2	35	M	22.5	C3-C4	HEICS	0
3	81	F	25.1	C5-C6	DH	7
4	29	F	23.7	C6-C7	HEICS	0
5	78	M	22.4	C5-C6	DH	6
6	40	M	23.1	C5-C6	HEICS	0

### 2.2 Total RNA extraction from cartilage endplate tissue

Total RNA was extracted from the tissues using TRIzol (Invitrogen, Carlsbad, CA, USA) according to the manual instructions. For tissue samples, approximately 60 mg of tissue was ground into a powder using liquid nitrogen, and the powdered samples were transferred into a 2-ml tube containing 1.5 ml of TRIzol reagent. The mixture was then centrifuged at 12,000 × *g* for 5 min at 4°C. The supernatant was transferred to a new 2.0-ml tube, to which was added 0.3 ml of chloroform/isoamyl alcohol (24:1) per 1.5 ml of TRIzol reagent. After the mixture was centrifuged at 12,000 × *g* for 10 min at 4°C, the aqueous phase was transferred to a new 1.5-mL tube, to which was added an equal volume of supernatant of isopropyl alcohol. The mixture was then centrifuged at 12,000 × *g* for 20 min at 4°C, and the supernatant was removed. After being washed with 1 ml of 75% ethanol, the RNA pellet was air-dried in the biosafety cabinet and then dissolved by adding 25–100 μl of DEPC-treated water. Subsequently, total RNA was qualified and quantified using a NanoDrop and Agilent 2100 Bioanalyzer (Thermo Fisher Scientific, MA, USA).

### 2.3 NGS

Libraries were prepared using 1 μg total RNA for each sample. Total RNA was purified through electrophoretic separation on a 15% urea denaturing polyacrylamide gel electrophoresis (PAGE) gel. The small RNA regions corresponding to the 18–30 nt bands in the marker lane (14–30 ssRNA Ladder Marker, TAKARA) were excised and recovered. Then, the 18–30 nt small RNAs were ligated to adenylated 3′ adapters annealed to unique molecular identifiers (UMIs), followed by the ligation of 5’adapters. The adapter-ligated small RNAs were subsequently transcribed into cDNA using SuperScript II Reverse Transcriptase (Invitrogen, USA), and then several rounds of PCR amplification using PCR Primer Cocktail and PCR Mix were performed to enrich the cDNA fragments. The PCR products were selected through agarose gel electrophoresis with target fragments 110–130 bp and then purified using a QIAquick Gel Extraction Kit (QIAGEN, Valencia, CA). The libraries were assessed for quality and quantity using two methods: the distribution of the fragment size was checked using the Agilent 2,100 Bioanalyzer, and the libraries were quantified using real-time quantitative PCR (qPCR) with TaqMan probes. The final ligation PCR products were sequenced using the BGISEQ-500 platform (BGI-Shenzhen, China).

### 2.4 Real-time quantitative PCR

Six pairs of frozen cartilage endplate tissue samples were divided into two groups, of which three samples were in the normal group (normal) and the other three samples were in the degeneration group (model). Then, total RNA from the samples was isolated and purified using TRIzol (Ambion) reagent following the manufacturer’s instructions. The extracted RNA was then tested for concentration using NanoPhotometer N50. Total RNA was reverse-transcribed into cDNA using the SweScript RT I First Strand cDNA Synthesis Kit(Servicebio). Reverse transcription product cDNA was diluted 5–20 times with ddH2O (RNase/DNase free). Subsequently, polymerase chain reaction (PCR) amplification reaction was performed using CFX96 real-time quantitative PCR instrument. Each qRT-PCR reaction was performed in triplicate as follows: step 1: denaturation at 95°C for 10 min; step 2: 40 cycles of 95°C for 15 s and 60°C for 1 min.The above reactions were subjected to 40 cycles. Primer sequences are shown in Table 2.

**Table 2:** The Primer Sequences of Diagnostic Genes.

Primer	Sequence
miR-25-3p	Forward: CATTGCACTTGTCTCGGTCTGA
	Reverse: CTGTCAACGATACGCTACGTAACG
miR-345-5p	Forward: GCTGACTCCTAGTCCA
	Reverse: TGGTGTCGTGGAGTCG
GRPDH	Forward: CATCATCAGCAGGGTGTGGC
	Reverse: CCCAATGCGGTAGGACAC

### 2.5 Western blot

Cartilage endplates were lysed with RIPA lysis buffer containing protease inhibitors (Beyotime, Shanghai, China). Proteins (20 μg per lane) were separated using 10% SDS polyacrylamide gel electrophoresis (PAGE) and then transferred to activated polyvinylidene fluoride (PVDF) membranes. After being blocked with 5% skimmed milk, the PVDF membranes were incubated with primary antibodies overnight at 4°C. The primary antibodies were BRD4 (1:1000, Proteintech, 28486-1-AP), BECN1 (1:1000, Proteintech, 11306-1-AP), and GAPDH (1,2000, Proteintech, 60004-1-AP). The blots were then incubated with appropriate secondary antibodies for 2 h at room temperature, after which protein bands were detected using an ECL chemiluminescence kit (EMD Merck Millipore, Darmstadt, Germany) and a chemiluminescence system (Bio-Rad Laboratories, Hercules, CA, USA).

### 2.6 Analysis

The raw sequencing data are called raw tags. The raw tags were processed using the following steps: low-quality tags were removed; tags with five primer contaminants were removed; tags without three primers were removed; tags without insertion were removed; tags with poly A were removed; tags shorter than 18 nt were removed. After filtering, the clean tags were mapped to the reference genome and other sRNA databases, including miRbase, siRNA, piRNA, and snoRNA, with Bowtie2. Particularly, cmsearch was performed for Rfam mapping. The software miRDeep2 was used to predict novel miRNAs by exploring the secondary structure, and Piano was used to predict piRNAs. RNAhybrid, miRanda, and TargetScan were used to predict the target genes of miRNAs. The small RNA expression level was calculated by counting absolute numbers of molecules using unique molecular identifiers. Differential expression analysis was performed using DEGseq, Q-value ≤ 0.001, and the absolute value of Log2Ratio ≥ 1 as the default threshold to determine the significance of expression difference. To annotate gene functions, all target genes were aligned against the Kyoto Encyclopedia of Genes (KEGG) and Gene Ontology (GO) databases. The GO enrichment and KEGG enrichment analyses of the target genes were performed using phyper, a function of R. The *p*-value was corrected using the Bonferroni method, and a corrected *p*-value ≤0.05 was taken as a threshold. GO terms or KEGG terms fulfilling this condition were defined as significantly enriched terms. All data were presented as mean ± SD and analyzed using GraphPad Prism (version 8.3.0) and R (version 4.2.1).

## 3 Result

### 3.1 Distribution of sample expression

**Figure 1:**
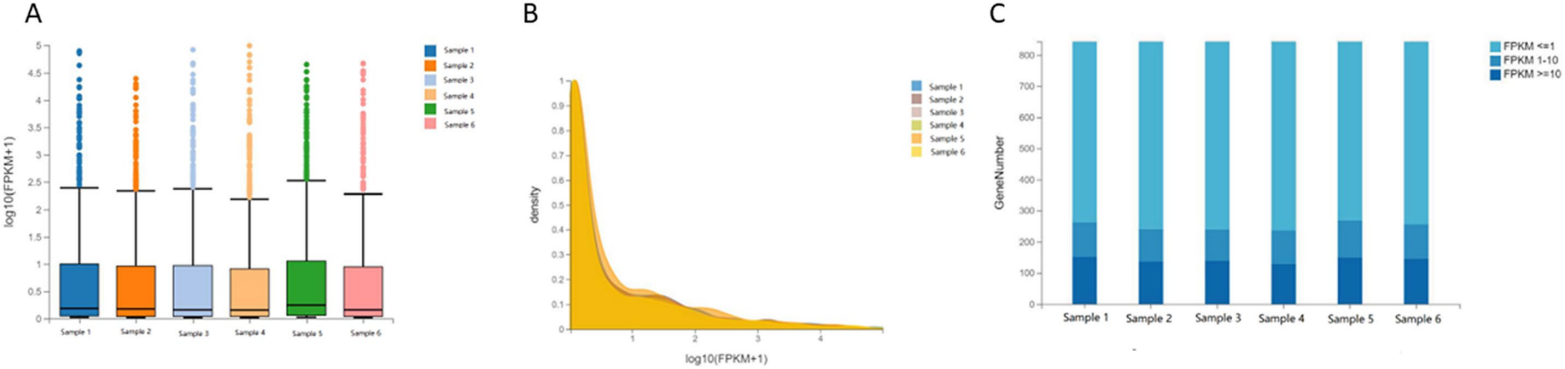
Distribution of sample expression. **(A)** Boxplot of gene expression levels in human cartilage endplate. **(B)** Densityplot of gene expression levels in human cartilage endplate. **(C)** Stacked Bar Chart of gene expression levels in human cartilage endplate.

The distribution of expression levels in human cartilage endplate tissues was shown through boxplots, and the trend of gene abundance in the samples with the change of expression levels was shown through density plots. To further intuitively display the genes of each sample in different FPKM intervals, the number of genes in the three levels of FPKM (FPKM<=1, FPKM 1–10, and FPKM> = 10) was analyzed, respectively. It was shown that there was no significant difference in the dispersion and concentration of gene expression levels in each sample and the number of genes in different FPKM intervals (Figure 1).

### 3.2 MicroRNAs differentially expressed in degenerated endplate chondrocytes

To find the differences in mRNA expression between the normal intervertebral disk and the cartilage endplate of the degenerated intervertebral disk, we screened all differentially expressed microRNAs more than four times between two groups according to their folding changes |log2FC| > =2 using next-generation sequencing. Compared to the control group, the experimental group had eight significant downregulated microRNAs, namely, hsa-miR-203b-5p, hsa-miR-323b-5p, hsa-miR-4423-5p, hsa-miR-141-3p, hsa-miR-205-5p, hsa-miR-3184-3p, hsa-miR-342-5p, and hsa-miR-203a-3, and 22 significant upregulated microRNAs, namely, hsa-miR-636, hsa-miR-103a-2-5p, hsa-miR-3151-5p, hsa-miR-550b-2-5p, hsa-miR-585-5p, hsa-miR-7976, hsa-miR-548aq-3p, hsa-miR-10395-3p, hsa-miR-10395-3p, hsa-miR-589-3p, hsa-miR-548av-3p, hsa-let-7b-3p, hsa-miR-363-3p, hsa-miR-624-5p, hsa-miR-665, hsa-miR-1307-5p, hsa-miR-200b-5p, hsa-miR-1249-3p, hsa-miR-25-3p, hsa-miR-103b, hsa-miR-345-5p, hsa-miR-301b-3p, and hsa-miR-200a-5p (Q ≤ 0.001, Table 3).

**Table 3:** The sequencing differential genes of human cartilage endplate.

	Gene ID	Type	log2 (Control/Treat)	Qvalue (Control-vs.-Treat)
Up	hsa-miR-203b-5p	miRNA	4.554937474	4.75E-04
	hsa-miR-323b-5p	miRNA	2.924171283	2.48E-05
	hsa-miR-4423-5p	miRNA	2.32047222	1.96E-08
	hsa-miR-141-3p	miRNA	4.282857928	5.17E-11
	hsa-miR-205-5p	miRNA	6.449755237	4.30E-25
	hsa-miR-3184-3p	miRNA	3.133136849	0
	hsa-miR-342-5p	miRNA	2.182968696	2.54E-49
	hsa-miR-203a-3p	miRNA	6.039177386	0
Down	hsa-miR-636	miRNA	−4.030025027	6.38E-04
	hsa-miR-103a-2-5p	miRNA	−3.351953122	3.32E-04
	hsa-miR-3151-5p	miRNA	−2.515451854	2.19E-04
	hsa-miR-550b-2-5p	miRNA	−5.145502244	1.58E-04
	hsa-miR-585-5p	miRNA	−5.252417448	8.08E-05
	hsa-miR-7976	miRNA	−3.18202812	4.09E-05
	hsa-miR-548aq-3p	miRNA	−2.073093749	2.31E-05
	hsa-miR-10395-3p	miRNA	−3.69299004	2.05E-05
	hsa-miR-589-3p	miRNA	−2.445062526	4.83E-06
	hsa-miR-548av-3p	miRNA	−3.252417448	2.07E-08
	hsa-let-7b-3p	miRNA	−2.053871769	8.30E-09
	hsa-miR-363-3p	miRNA	−2.18202812	2.74E-09
	hsa-miR-624-5p	miRNA	−2.118834294	6.28E-10
	hsa-miR-665	miRNA	−3.843611903	5.26E-29
	hsa-miR-1307-5p	miRNA	−3.795559773	1.32E-37
	hsa-miR-200b-5p	miRNA	−3.536985016	8.22E-38
	hsa-miR-1249-3p	miRNA	−2.907853157	4.84E-42
	hsa-miR-25-3p	miRNA	−2.380284755	0
	hsa-miR-103b	miRNA	−4.893779119	2.76E-255
	hsa-miR-345-5p	miRNA	−2.194304227	0
	hsa-miR-301b-3p	miRNA	−2.492368241	3.69E-61
	hsa-miR-200a-5p	miRNA	−3.5235645	1.49E-73

### 3.3 Association network analysis

**Figure 2:**
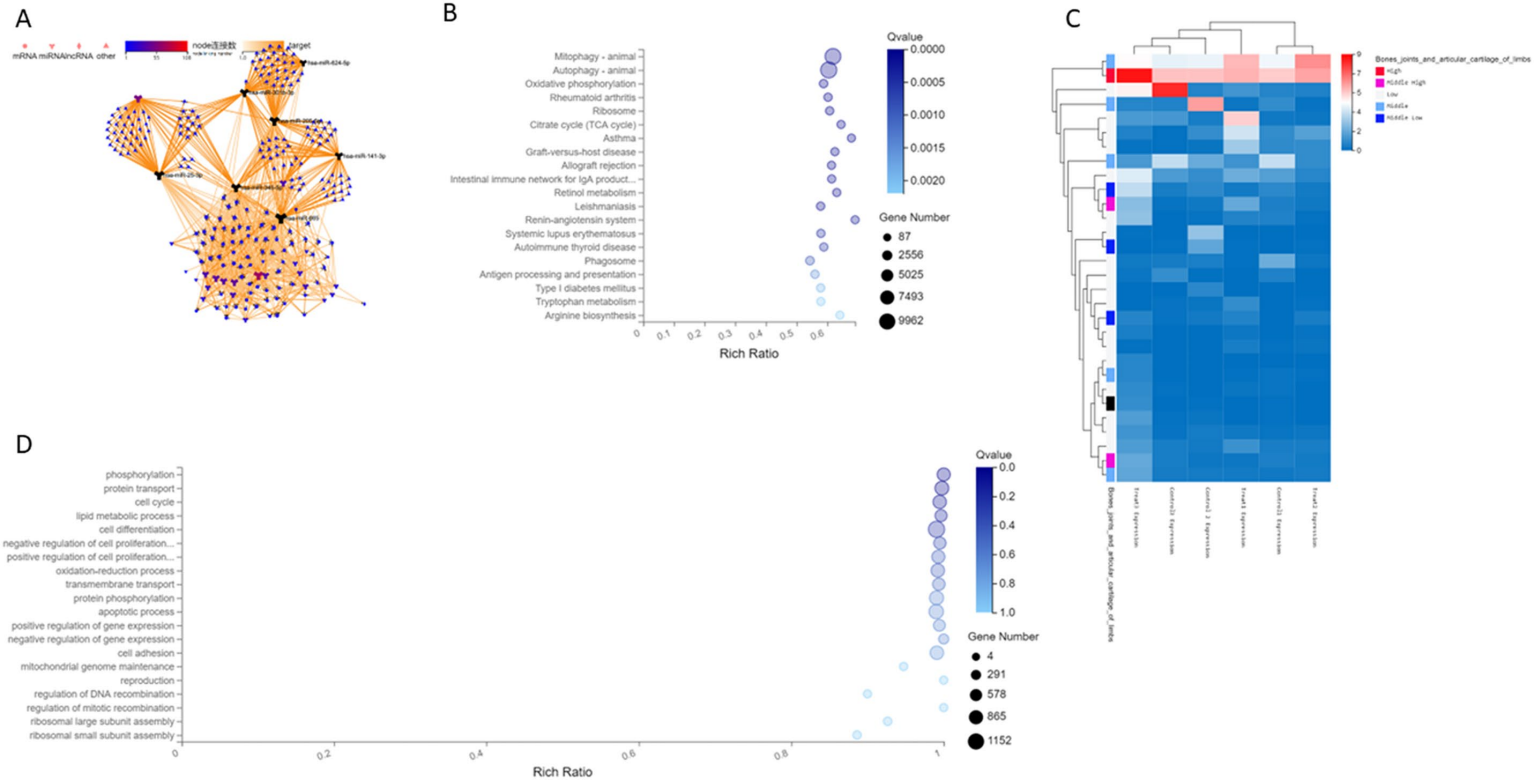
**(A)** The differential gene association network of human cartilage endplate. **(B)** A Histogram of differential genes of human cartilage endplate by KEGG sequencing. The X-axis is the ratio of enrichment [the formula is Ratio = Term Candidate Gene Num/Term Gene Num]. The Y-axis is KEGG Pathway. The size of the bubble represents the number of genes annotated on a certain KEGG Pathway, and the color represents the Q value. The darker the color, the smaller the Q value. **(C)** Clustering heat map of differential gene expression of human cartilage endplate by sequencing. **(D)** Enrichment bubble diagram of biological process of differential genes of human cartilage endplate by GO analysis. The X-axis is the ratio of enrichment [the formula is Ratio = Term Candidate Gene Num/Term Gene Num]. The Y-axis is GO Term. The size of the bubble represents the number of genes annotated on a GO Term, and the color represents the Q value. The darker the color, the smaller the Q value.

To further explore the relationship between differential microRNAs and their target genes, we performed target transformation on all differential genes and then performed association network analysis to obtain an association network diagram (Figure 2A). MicroRNA hsa-miR-25-3p, hsa-miR-301b-3p, hsa-miR-141-3p, hsa-miR-624-5p, hsa-miR-665, hsa-miR-345-5p, and hsa-miR-205-5p with corresponding mRNA and CircRNA constitute CircRNA-miRNA-mRNA regulation network, indicating that these differential genes have further research value.

### 3.4 Cluster analysis

According to the results of differential microRNA analysis, the hierarchical clustering analysis was performed using the pheatmap function in R software. The horizontal axis represents the log2 of the sample (expression value +1), whereas the first column of the vertical axis represents the expression of genes in limb joints and articular cartilage in the public database. Red indicates high expression and blue indicates low expression. Follow-up is the expression of this gene in cartilage endplate tissues according to the sequencing results. Our results showed that the expression of differential genes in the cartilage endplates was similar to that in the public database, indicating the reliability of the sequencing (Figure 2C). Moreover, hsa-miR-25-3p and hsa-miR-345-5p had significant differences in expression between degenerated human cartilage endplate tissues and normal human cartilage endplate tissues.

### 3.5 Prediction and analysis of differential microRNA target protein

Through prediction and intersection of target protein in the three public databases, namely, miRanda,^1^ TargetScan,^2^ and RNAhybrid,^3^ we found that the significant target proteins of miR-25-3p and miR-345-5p were BRD4 and BECN1, respectively.

Analysis based on the KEGG pathway helps us to further understand the biological function of genes. The KEGG pathway is the main public database on pathways. Pathway significant enrichment analysis takes the KEGG pathway as the unit to find pathways that are significantly enriched in candidate genes compared to the whole-genome background and through hypergeometric test. Pathway significant enrichment can select the most important biochemical metabolic pathways and signal transduction pathways mediated by candidate genes. A total of 395 microRNAs with significantly regulated genes were identified as targets. Through the KEGG pathway analysis of these target genes, it was found that the differential microRNAs and target genes were enriched in different signaling pathways involved in mainly mitophagy and autophagy (Figure 2B).

Gene Ontology (GO) analysis can simply annotate gene products from three aspects, namely, functions, biological pathways, and cellular localization. Therefore, GO enrichment analysis provides a rough understanding of the biological functions, pathways, or cellular localizations in which the genes are enriched. Through GO analysis, it was found that the differential microRNAs and target genes were involved in different biological processes, such as metabolic processes, growth, localization, biological attachment, cell processes, and biological regulation. The top 20 biological processes enriched by differential microRNAs and target genes are as follows: phosphorylation, protein transport, cell cycle, lipid metabolism, cell differentiation, negative regulation of cell proliferation, positive regulation of cell proliferation, oxidation–reduction process, transmembrane transport, protein phosphorylation, apoptosis process, positive regulation of gene expression, negative regulation of gene expression, cell adhesion, mitochondrial genome maintenance, reproduction, regulation of DNA recombination, regulation of mitotic recombination, ribosomal large subunit assembly, and ribosome small subunit assembly (Figure 2D).

### 3.6 Validation of miRNAs and target proteins

**Figure 3:**
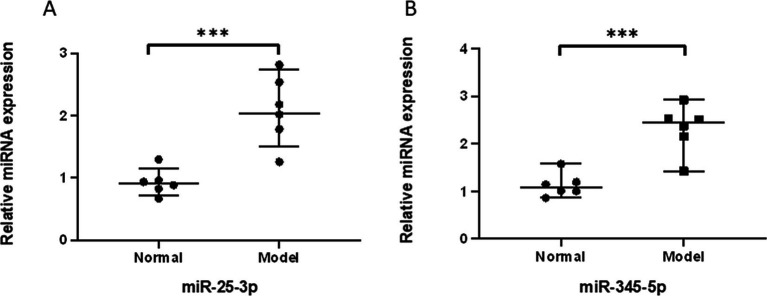
qPCR validation of the significantly differentially expressed miRNAs between Degeneration and normal controls. **(A)** qPCR validation of the miR-25-3p between Degeneration and normal controls. **(B)** qPCR validation of the miR-345-5p between Degeneration and normal controls.All data are reported as the mean ± s.d. Statistical significance was determined by Student’s t-test. ***p < 0.001.

**Figure 4:**
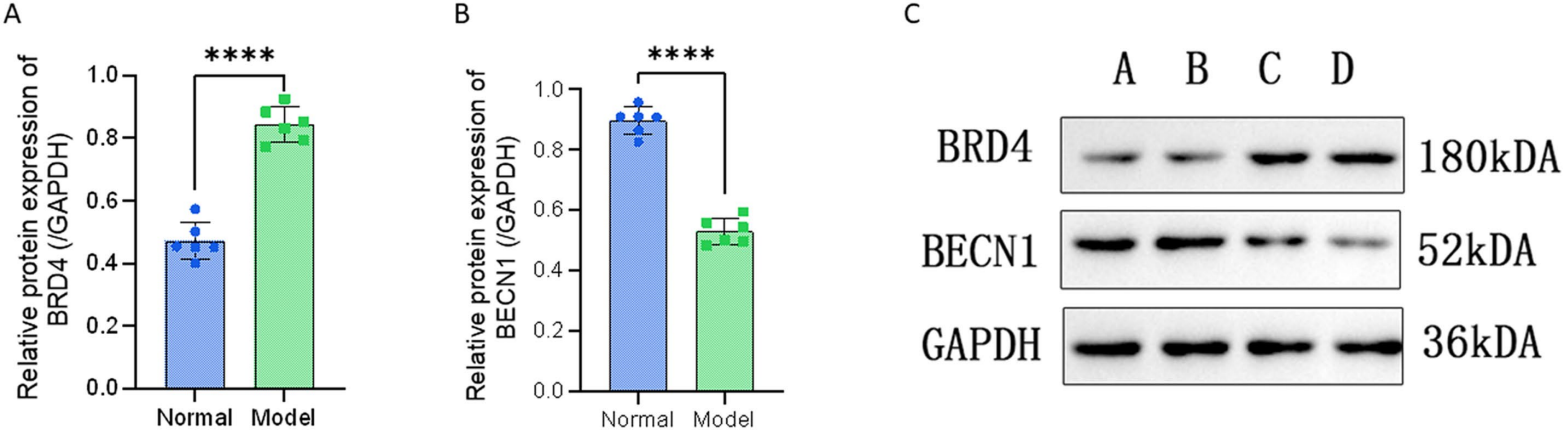
Western blot validation of the interest protein between Degeneration and normal controls. **(A)** Western blot validation of the BRD4 between Degeneration and normal controls. **(B)** Western blot validation of the BECN1 between Degeneration and normal controls. **(C)** Experimental bands for Western blot, A and B are normal cartilage endplate tissues, C and D are Degeneration cartilage endplate tissues. All data are reported as the mean ± s.d. Statistical significance was determined by Student’s t-test. ****p < 0.0001.

We performed validation of the selected miRNAs and their target protein through PCR and Western blot analysis. The amplification fold of miR-25-3p in the degenerative cartilage endplate tissue was 2.099 ± 0.552, while that in the normal cartilage endplate tissue was 0.928 ± 0.208. The *p*-value was 0.0007, indicating a statistically significant difference (*p* ≤ 0.001; Figure 3A). The amplification fold of miR-345-5p in the degenerative cartilage endplate tissue was 2.317 ± 0.506, while that in the normal cartilage endplate tissue was 1.133 ± 0.247. The *p*-value was 0.0004, indicating a statistically significant difference (*p* ≤ 0.001; Figure 3B). In addition, the Western blot analysis showed that the expression of BRD4 in the degenerative cartilage endplate was 0.844 ± 0.056, while that in the normal cartilage endplate tissue was 0.472 ± 0.059. The *p*-value was ≤0.0001, indicating a statistically significant difference (Figure 4A). The expression of BECN1 in the degenerative cartilage endplate was 0.530 ± 0.043, while that in the normal cartilage endplate tissue was 0.896 ± 0.045. The *p*-value was ≤0.0001, indicating a statistically significant difference (Figure 4B). These findings substantiate the reliability of our sequencing data that miR-25-3p and miR-345-5p were increased in the cartilaginous endplate (Figure 4C). In addition, the target protein BRD4 was found to be increased, while BECN1 showed a decrease in the degenerative cartilage endplate tissue.

## 4 Discussion

At present, many studies have reported that the increase in pro-inflammatory factors can lead to the change of expression of related microRNAs (10). Zhang et al. (11) reported that more severe degeneration of intervertebral disk tissue correlates with higher expression levels of inflammatory cytokines and lower levels of miR-140-5p. They found that overexpression of miR-140-5p can inhibit degeneration caused by intervertebral disk inflammation by downregulating Toll-like receptor 4. In addition, it was found that changes in the expression of microRNAs also altered the secretion of pro-inflammatory cytokines. Feng et al. (12) found that overexpression of miR-146a could significantly reduce the levels of pro-inflammatory factors in nucleus pulposus cells through the TARF6/NF-kB pathway. In a recent study, miR-181a was found to contribute anti-inflammatory effects by inhibiting the ERK pathway in mice with intervertebral disk degeneration (13). In this study, cartilage endplate cell degeneration was induced by the inflammatory factor TNFα, and high-throughput sequencing also showed that the inflammatory response would lead to changes in the expression of cellular microRNAs. Among them, eight microRNAs were significantly downregulated, namely, hsa-miR-203b-5p, hsa-miR-323b-5p, hsa-miR-4423-5p, hsa-miR-141-3p, hsa-miR-205-5p, hsa-miR-3184-3p, hsa-miR-342-5p, and hsa-miR-203a-3p. In addition, 22 microRNAs were significantly upregulated, namely, hsa-miR-636, hsa-miR-103a-2-5p, hsa-miR-3151-5p, hsa-miR-550b-2-5p, hsa-miR-585-5p, hsa-miR-7976, hsa-miR-548aq-3p, hsa-miR-10395-3p, hsa-miR-10395-3p, hsa-miR-589-3p, hsa-miR-548av-3p, hsa-let-7b-3p, hsa-miR-363-3p, hsa-miR-624-5p, hsa-miR-665, hsa-miR-1307-5p, hsa-miR-200b-5p, hsa-miR-1249-3p, hsa-miR-25-3p, hsa-miR-103b, hsa-miR-345-5p, hsa-miR-301b-3p, and hsa-miR-200a-5p. However, because the significantly different microRNAs screened in this study were defined as those exhibiting a difference of more than four times, the microRNAs mentioned in the abovementioned studies were not included in our screening.

Current studies have shown that autophagy is closely related to the pathogenesis of osteoarthritis. Roach et al. (14) first reported that autophagy contributes to cell death in articular cartilage, termed osteomalacia. Caramés et al. (15) confirmed that the occurrence of senile osteoarthritis is related to the decreased expression of Unc-51-like kinase 1 (ULK1), Beclin-1, and microtubule-associated protein 1 light chain 3 (LC3) in articular cartilage and speculated that autophagy may protect chondrocytes from death. In addition, it has been proposed that hypoxia-inducible factor (HIF)-2α can inhibit the autophagy of chondrocytes (16). It is generally accepted that autophagy plays a dual role and is involved in cell survival and death (17, 18). Autophagy also has dual effects on chondrocytes in osteoarthritis. Autophagy is downregulated in aged articular cartilage chondrocytes, but the expression of autophagy-related proteins is not reduced in osteoarthritis. Moreover, it is found that excessive autophagy can lead to the death of cartilage cells in the bones and joints, resulting in the occurrence of osteoarthritis (19). Hiroshi et al. (20) found that the LC3-II and Beclin-1 levels are higher in chondrocytes in osteoarthritis than in normal cartilage using Western blot analysis. They reported that inhibition of autophagy enhances IL-1β-induced changes in osteoarthritis-related genes, while activation of autophagy can inhibit the occurrence of osteoarthritis by removing ROS from damaged mitochondria.

Autophagy plays an important physiological and pathological role in intervertebral disk degeneration. In clinical cases, autophagy levels in cartilage endplate cells were found to be significantly downregulated in cervical spondylosis patients compared to those with cervical spine fractures and dislocations (21). On the contrary, autophagy is upregulated in annulus fibrosus cells of patients with lumbar disk degeneration compared to normal annulus fibrosus cells (22). Yang et al. found that autophagy protects nucleus pulposus cells from apoptosis induced by cyclic mechanical stress (23). It was reported that under normal physiological conditions, a low level of autophagy was found in nucleus pulposus (NP) and annulus fibrosus (AF) cells isolated from normal adult rats, indicating that autophagy has a role in maintaining normal integrity and activity of the intervertebral disk cell (24). However, autophagy was significantly increased in NP and AF cells of degenerated rats (25, 26). Furthermore, it has been reported that upregulation of autophagy in rat cartilage endplates can reduce oxidative stress-induced damage to cartilage endplate cells (27). This study clarified that autophagy plays an important role in cartilage endplate degeneration, which proved the accuracy of this sequencing.

In a recent study, Sakamaki et al. (28) revealed that BRD4 can inhibit autophagy by negatively regulating the expression of several autophagy and lysosomal genes at the transcription level, thereby modulating lysosomal gene expression and lysosomal function. Wen et al. (29) also found that BRD4 is a novel transcriptional regulator inhibiting autophagy and lysosomal function. Regarding its role in the intervertebral disk, the latest study found that inhibition of BRD4 with or without the involvement of TNF-α can enhance autophagy and reduce the degradation of the extracellular matrix, and inhibition of BRD4 can inhibit the activation of NLRP3 and NF-κB signaling pathway in rat nucleus pulposus cells. In addition, inhibition of autophagy promotes NLRP3-mediated pyrophosphorylation, which ultimately exacerbates extracellular matrix degradation in nucleus pulposus cells. Furthermore, inhibition of BRD4 also inhibits NF-κB signaling and TNF-α-induced NLRP3 activity and attenuates cell apoptosis (30), which further proves the reliability of this study. Chen Z et al. created an inflammatory degeneration model by treating chondrocytes with TNF-α and found that the overexpression of miR-30b downregulates autophagy genes and upregulates the expression of pro-apoptotic genes, while inhibition of miR-30b enhances autophagy and alleviates chondrocyte degradation, which plays a protective role in inflammation-induced osteoarthritis (31). In this study, BRD4 was significantly upregulated in degenerated cartilage endplate, while the autophagy-regulating protein BECN1 was significantly downregulated, which indicated that the interaction of miR-25-3p and miR-345-5p and their target proteins in the process of inflammatory cartilage endplate degeneration downregulates autophagy of cartilage endplate leading to the degeneration.

The limitations of this study include the small sample size and the lack of additional animal experiments. The future validation of miR-25-3p and miR-345-5p’s involvement in cartilage endplate degeneration can be further substantiated through the modulation of Mir-25-3p and Mir-345-5p expression, as well as the inhibition of BRD4 and BECN1 expression levels within the context of cartilage endplate degeneration.

## 5 Conclusion

In this study, we found the genes with significant differences between normal and degenerated cartilage endplates through sequencing, among which miR-25-3p, miR-345-5p, and their target genes BRD4 and BECN1 were all involved in autophagy and had great research value. In conclusion, this study provides novel therapeutic strategies for the diagnosis and treatment of intervertebral disk degeneration.

## Data Availability

The original contributions presented in the study are included in the article, further inquiries can be directed to the corresponding author/s.

## Footnotes

^1^http://www.microrna.org/microrna/home.do

^2^http://www.targetscan.org/vert_72/

^3^https://bibiserv.cebitecuni-bielefeld.de/rnahybrid
